# The cement of the tube-dwelling polychaete *Sabellaria alveolata*: a complex composite adhesive material

**DOI:** 10.3762/bjnano.16.138

**Published:** 2025-11-11

**Authors:** Emilie Duthoo, Aurélie Lambert, Pierre Becker, Carla Pugliese, Jean-Marc Baele, Arnaud Delfairière, Matthew J Harrington, Patrick Flammang

**Affiliations:** 1 Biology of Marine Organisms and Biomimetics Unit, Research Institute for Biosciences, University of Mons, Place du Parc 23, B-7000 Mons, Belgium; 2 Department of Geology, Faculty of Engineering, University of Mons, 7000 Mons, Belgium; 3 Department of Chemistry, McGill University, 801 Sherbrooke Street West, Montreal, Quebec H3A 0B8, Canada

**Keywords:** adhesive protein, Annelida, biological material, Polychaeta, protein phosphorylation

## Abstract

Adhesives produced by marine organisms offer remarkable performance and serve as a major source of inspiration for developing biomimetic adhesives. However, a thorough understanding of their composition and operating mechanism is essential for advancing such applications. Sabellariid tubeworms are model organisms in bioadhesion research, and their adhesive system has been characterized in several studies. However, some aspects of cement formation are still poorly understood and several differences have been pointed out between the two main model species. This study aims to investigate the adhesive system of *Sabellaria alveolata* by identifying new potential adhesive proteins, as well as describing the ultrastructure and elemental composition of the cement cells and their secretion. Different adhesive proteins are packaged in one or the other of two types of cement cells, namely, those containing homogeneous granules and those containing heterogeneous granules with lamellar inclusions. Phosphoserine has been identified as one of the main modified amino acids in tubeworm cement and, using in situ hybridization, we propose that FAM20C kinases would be the enzymes responsible for the phosphorylation of serine residues in adhesive proteins. Comparison between the ultrastructure of the granules and that of the cement suggests that the inclusions of the heterogeneous granules would inflate through a still unexplained process to form hollow spheroids dispersed in the cement matrix, leading to the formation of a complex composite material.

## Introduction

Many invertebrate marine organisms have adhesive mechanisms that allow them to firmly attach to various substrates in a wet and salty environment [1–2]. This remarkable ability has raised the interest of scientists in developing bio-inspired underwater adhesive materials for various applications, particularly in the industrial and biomedical fields [3–4]. Polychaetes of the family Sabellariidae are one of the model organisms that have been studied extensively for their adhesion and have fascinated researchers since the 18th century [5]. Two species in particular have been the subject of numerous studies focused on the microstructure and composition of their adhesive secretion, namely, the North American species *Phragmatopoma californica* Krøyer in Mörch, 1863 and the European species *Sabellaria alveolata* (Linnaeus, 1767). Sabellariids are tube-dwelling worms that build their tube using a specialized building organ located near the mouth. The two finger-like lobes of this organ allow them to manipulate sand grains or shell fragments and to glue them together with several spots of a strong proteinaceous cement [6–9]. The building organ is the external part of an extended glandular system comprising two types of cement cells located in the parathoracic region of the worm, around the digestive tract and at the base of parapodia. The two types of cells can be distinguished by the morphology of their secretory granules, which are either homogeneous or heterogeneous containing inclusions [6,10–12]. The adhesive proteins are packaged into secretory granules via a process called complex coacervation, which involves the aggregation of oppositely charged proteins along with a sulfated polysaccharide and significant amounts of Mg^2+^ and Ca^2+^ ions [8–9,12–13]. The two types of secretory granules are secreted separately and intact, but rapidly fuse to form a porous cement spot whose pores would derive from the heterogeneous granule inclusions [9,12–13].

In *P. californica*, the cement would consist of up to 25 proteins, but only five, referred to as Pc-1 to -5, have been partially characterized [8,12,14–16]. Pc-1 and Pc-2 are basic proteins that contain glycine-rich peptide repeats [14–15]. A fraction of their tyrosine residues are post-translationally hydroxylated to form 3,4-dihydroxyphenylalanine (DOPA) residues, which may facilitate bonding to mineral surfaces and play a role in quinone-mediated cross-linking during cement hardening [14–15]. Pc-3 exists in at least two major isoforms, Pc-3A and Pc-3B. Both isoforms are exceptionally rich in serine (72.9 mol %), with up to 90% of these residues undergoing post-translational phosphorylation [15]. As a result, Pc-3 is an unusually acidic protein. Pc-4 and Pc-5 are histidine-rich basic proteins. In the adhesive secretion of *S. alveolata*, only three adhesive proteins have been identified [17], although a differential transcriptomic study suggested the existence of many others [18]. The proteins Sa-1, Sa-2, Sa-3A, and Sa-3B share the same physico-chemical characteristics as their homologues in *P. californica* [17]. In both species, the polyphosphorylated proteins appear to be segregated exclusively in the inclusions within the heterogeneous granules [9,17].

Despite the remarkable abundance of phosphoserine (pSer) residues in the Sabellariid worm adhesive system, the identity of the kinase involved in the maturation of adhesive proteins is not well understood. Sagert et al. [19] proposed that the phosphorylation of cement proteins is catalyzed by a casein kinase, but its sequence could not be retrieved [12,16]. Since then, casein kinases have been identified as FAM20C kinases [20–22]. FAM20C is a secreted protein that is responsible for phosphorylating S-x-E/pS motifs but also polyserine stretches within proteins in the secretory pathway [20,23]. It is involved in various biological processes, including mineral formation as it phosphorylates extracellular proteins that regulate biomineralization [20,24]. This enzyme could therefore be a candidate kinase for the modification of adhesive proteins in *S. alveolata*.

This study aims at better characterization of the adhesive system of *S. alveolata* through the ultrastructural and chemical characterization of the two types of adhesive cells and the cement they produce, as well as the identification of new adhesive protein candidates. Another goal is to address the gap in knowledge about adhesive protein maturation by identifying and localizing the kinases phosphorylating adhesive proteins using in silico analyses and in situ hybridization techniques. The results may provide new insights into the composition and biosynthesis of the adhesive secretion, which is crucial to the honeycomb worm’s survival.

## Methods

### Collection of honeycomb worms and samples preparation

Reef fragments of *S. alveolata* were collected at low tide in Champeaux, Bay of Mont Saint-Michel, France (48°43′50″N, 01°33′05″W). Additionally, some reef fragments were obtained from the Biological Sample Collection Service of the Station Biologique de Roscoff in Brittany, France. Animals were transported to the laboratory of Biology of Marine Organisms and Biomimetics (University of Mons, Belgium), where they were kept in a re-circulating aquarium chilled at 13 °C and filled with artificial seawater of 33 psu salinity. Animals used in our experiments were maintained and treated in compliance with the guidelines specified by the Belgian Ministry of Trade and Agriculture.

Individual tubes containing an individual worm were isolated from the reef fragment and placed in a petri dish. The distal third of each tube was then sectioned, fixed in 4% paraformaldehyde in phosphate-buffered saline, rinsed, and air-dried. Worms were left in the remaining proximal part of the tube and were provided with glass beads (425–600 µm in diameter; Sigma) to reconstruct the missing part [25].

### Scanning electron microscopy and elemental composition analyses

For secondary electron imaging, the anterior parts of a few worms as well as some reconstructed tube fragments were fixed in Bouin’s fluid for 24 h, dehydrated in graded ethanol, dried by the critical-point method, and mounted on aluminium stubs using carbon adhesive tabs. The samples were then coated with gold–palladium in a sputter-coater and observed using a JEOL JSM-7200F field-emission scanning electron microscope.

To observe the organization of natural tubes, air-dried tube fragments were placed vertically in 2.5 cm cylindrical brass molds, and embedded with petrographic epoxy resin (Hillquist inc., USA). After curing for 1 h at 80 °C, a transverse section of the mounted tubes was finely ground with SiC grit 800 abrasive suspensions on a high-flatness cast-iron lapping plate. The sections were then polished in three steps on textile cloths soaked with diamond suspensions of 6, 3, and 1 µm, respectively. Transverse sections through the tubes could be imaged with high resolution in SEM (JEOL JSM-7200F), showing the arrangement of cement spots binding mineral particles together. The epoxy resin embedding technique provided excellent preservation of the cement spot structure. Honeycomb worms embedded in Spurr resin (TEM samples) were used for the observation of cement gland secretory granules. All SEM images were acquired in low vacuum mode (50 Pa), with the backscattered electron detector.

X-ray microanalysis and elemental mapping were performed using an Oxford X-Max^N^ energy-dispersive spectrometer (EDS) equipped with an 80 mm^2^ silicon drift detector. Acquisition conditions on the SEM were 15 kV, 10 mm working distance, and 10 s live time acquisition at approximately 30–40% dead time. The spectra were acquired with an AZtec (Oxford Instrument) EDS data processing software.

### Transmission electron microscopy

The anterior part of *S. alveolata* individuals and single glass beads bearing cement spots were fixed for 3 h at 4 °C in a solution of 3% glutaraldehyde in cacodylate buffer (0.1 M, pH 7.8; osmolarity adjusted to 1030 mOsm·L^−1^ with NaCl). They were then rinsed three times for 10 min in a solution of cacodylate buffer (0.2 M, pH 7.8, adjusted to 1030 mOsm·L^−1^), and post-fixed for 1 h in 1% osmium tetroxide in cacodylate buffer (0.1 M, pH 7.8, adjusted to 1030 mOsm·L^−1^) in the dark. After a final rinse in cacodylate buffer, the cement spots were decalcified for 24 h in a 10% EDTA solution (pH ≈8). All the samples were then dehydrated in a series of ethanol baths of increasing strength (25%, 50%, 70%, 90%, and 100%) and embedded in Spurr resin. Semi-thin sections of 1 µm thickness were cut using a Reichert Om U2 ultramicrotome. They were then stained with a 1:1 mixture of 1% aqueous solution of methylene blue in 1% sodium tetraborate and 1% aqueous solution of azur II. Ultrathin sections, 70 nm thick, were then obtained using a Leica Ultracut UCT ultramicrotome fitted with a diamond knife. These sections were contrasted with uranyl acetate and lead citrate and observed using a Zeiss LEO 906E transmission electron microscope.

#### Identification and characterization of new adhesive protein and kinase candidates

Local basic local alignment search tool (BLAST) searches were performed in the transcriptome of the anterior part of *S. alveolata* [26] using the adhesive protein sequences of *P. californica* from the study by Endrizzi and Stewart [16] as queries. Additional searches were also performed using different FAM20C sequences retrieved from the NCBI database (NCBI accession numbers: AVI57681.1 (*Pinctada fucata*), XP_033744735.1 (*Pecten maximus*), CAD7192288.1 (*Sepia pharaonic*), XP_035824787.1 (*Aplysia californica*), Q5MJS3.1 (*Mus musculus*)*,* and Q8IXL6.2 (*Homo sapiens*)) (retrieved in January 2021).

All the obtained transcripts, as well as previously identified adhesive protein sequences from *S. alveolata* (NCBI accession numbers: Sa-1 – HE599563; Sa-2 – HE599584; Sa-3A – HE599605; Sa-3B – HE599626), were translated and analyzed in silico. Molecular weight and theoretical pI were computed using the ProtParam tool (https://web.expasy.org/protparam/) [27], and amino acid composition was analyzed using SAPS (https://www.ebi.ac.uk/jdispatcher/seqstats/saps) [28]. The presence of a signal peptide was predicted using SignalP 6.0 (https://services.healthtech.dtu.dk/services/SignalP-6.0/) [29]. Finally, the sequences were used in a reciprocal tBLASTn search against the NCBI non-redundant protein database to confirm identification.

#### Total RNA extraction and cDNA construction

Total RNA was extracted from different parts of three honeycomb worms using TRIzol^TM^ Reagent kit (Thermofisher). The parts selected were the head, parathoracic, abdominal, and caudal regions. Concentration and purity of the extracted RNA were measured with a UV–vis spectrophotometer (DENOVIX DS-11). A cDNA library was synthesized from the RNA extracted by reverse transcription polymerase chain reaction (RT-PCR) using the Reverse transcription kit (Roche).

#### Amplification by PCR

Double-stranded DNA templates were amplified by PCR using the Q5 High-Fidelity DNA Polymerase kit method (New England BioLabs), with primer designed by Open Primer 3 (bioinfo.ut.ee/primer3/) with an optimal amplicon length between 700 and 900 bp (Supporting Information File 1, Table S4). For the previously reported adhesive proteins, the primers were designed using the first clone of each cement precursor protein available on NCBI [17]. For in situ hybridization probe synthesis, a second PCR was done with a T7 promoter binding site (5′-GGATCCTAATACGACTCACTATAGG-3′) added to reverse strand PCR primers. After quality and size check by gel electrophoresis, PCR products were purified using the Wizard SV Gel and PCR clean-up system kit (Promega). The purified products were used for RNA probe synthesis after sequencing to check if the amplified sequence corresponds to the desired transcript.

#### Localization of the candidates using in situ hybridization

A few worms were retrieved from their tubes, and their anterior part was dissected and fixed in a 4% paraformaldehyde solution in phosphate-buffered saline (pH 7.4). The samples were then dehydrated through graded ethanol series and embedded in paraffin wax. Sections of 14 µm in thickness were cut with a Microm HM 340 E microtome and mounted on Superfrost Ultra Plus (Thermo Scientific) microscope slides using a Milli-Q water drop.

Antisense digoxigenin (DIG)-labelled RNA probes were synthesized with DIG RNA Labelling Kit (Roche) with T7 RNA polymerase and DIG–dUTP. In situ hybridization was performed according to the protocol of Lengerer and colleagues [30]. The RNA probes were used at a concentration of 0.2 ng·µL^−1^ on dewaxed sections of *S. alveolata* and detected with anti digoxigenin-AP Fab fragments (Roche) at a dilution of 1:2000. The signal was developed using the NBT/BCIP substrate (Roche) at 37 °C. The sections were observed using a Zeiss Axio Scope A1 light microscope with a 100× objective to distinguish both types of cement glands based on their secretory granule morphology, and images were taken with an AxioCam 305 digital camera (Carl Zeiss MicroImaging).

## Results

### Tube structure

**Figure 1:**
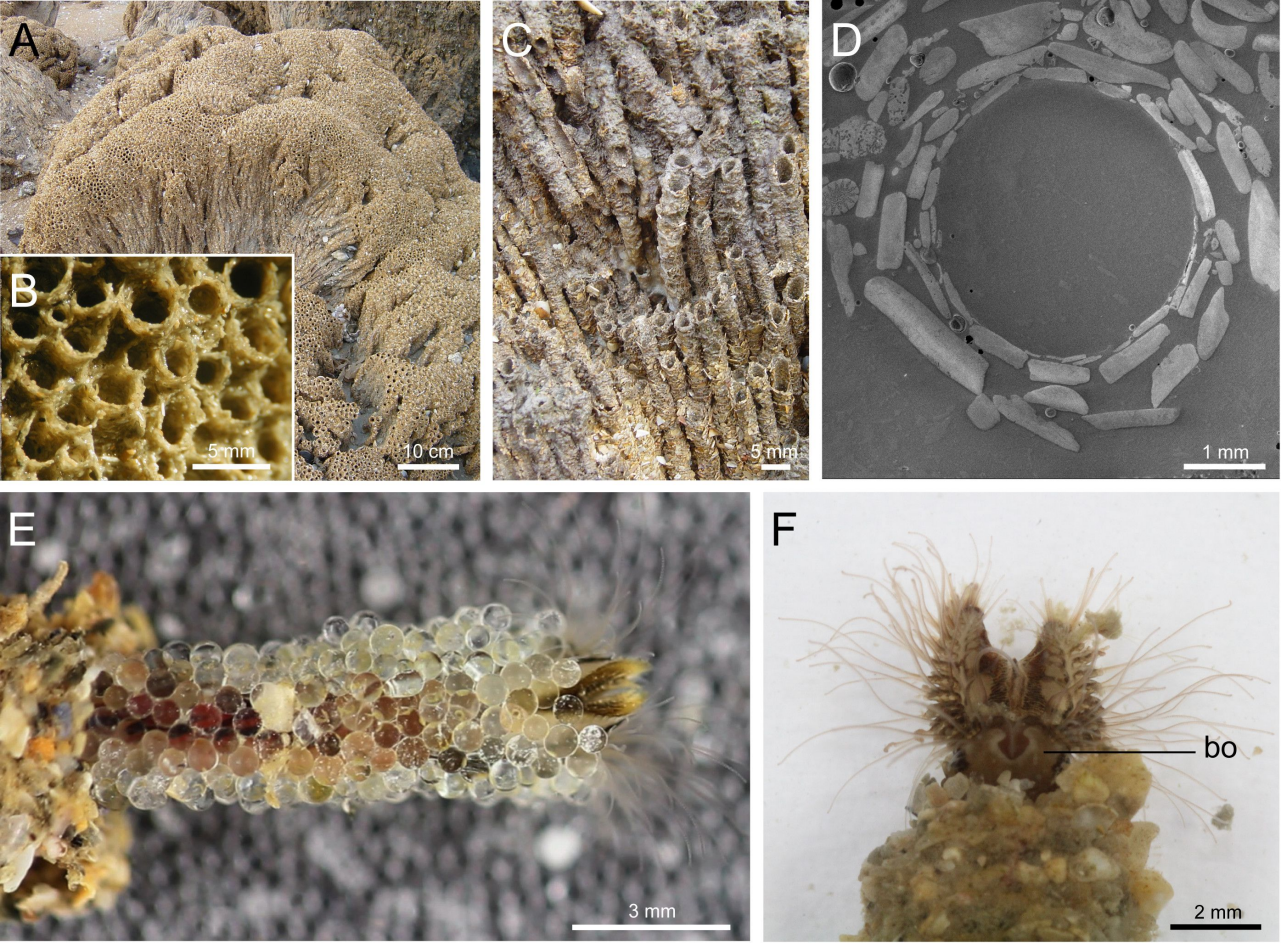
Structure of the tubes of *Sabellaria alveolata*. Picture of a reef fragment (Champeaux, Bay of Mont Saint-Michel) (A), with detailed views of the natural tubes (B, C). SEM image of an epoxy-embedded tube in cross section showing the arrangement of mineral particles (D). Individual of *S. alveolata* which extended its tube using provided glass beads (E). Another individual in its natural tube showing its building organ (F). Abbreviation: bo – building organ.

As described by Vovelle [6], the tubes of *S. alveolata* are generally rectilinear and cylindrical, measuring up to 12 cm in length (Figure 1A,C) and up to 4 mm in diameter (Figure 1B,D). These tubes are made up of sand grains and shell fragments arranged obliquely to the tube’s long axis in a funnel-like pattern, giving their upper part a flared appearance (Figure 1A,B). Internally, the tube is lined by a layer of flat mineral components covered with a thin, smooth organic layer (Figure 1D).

**Figure 2:**
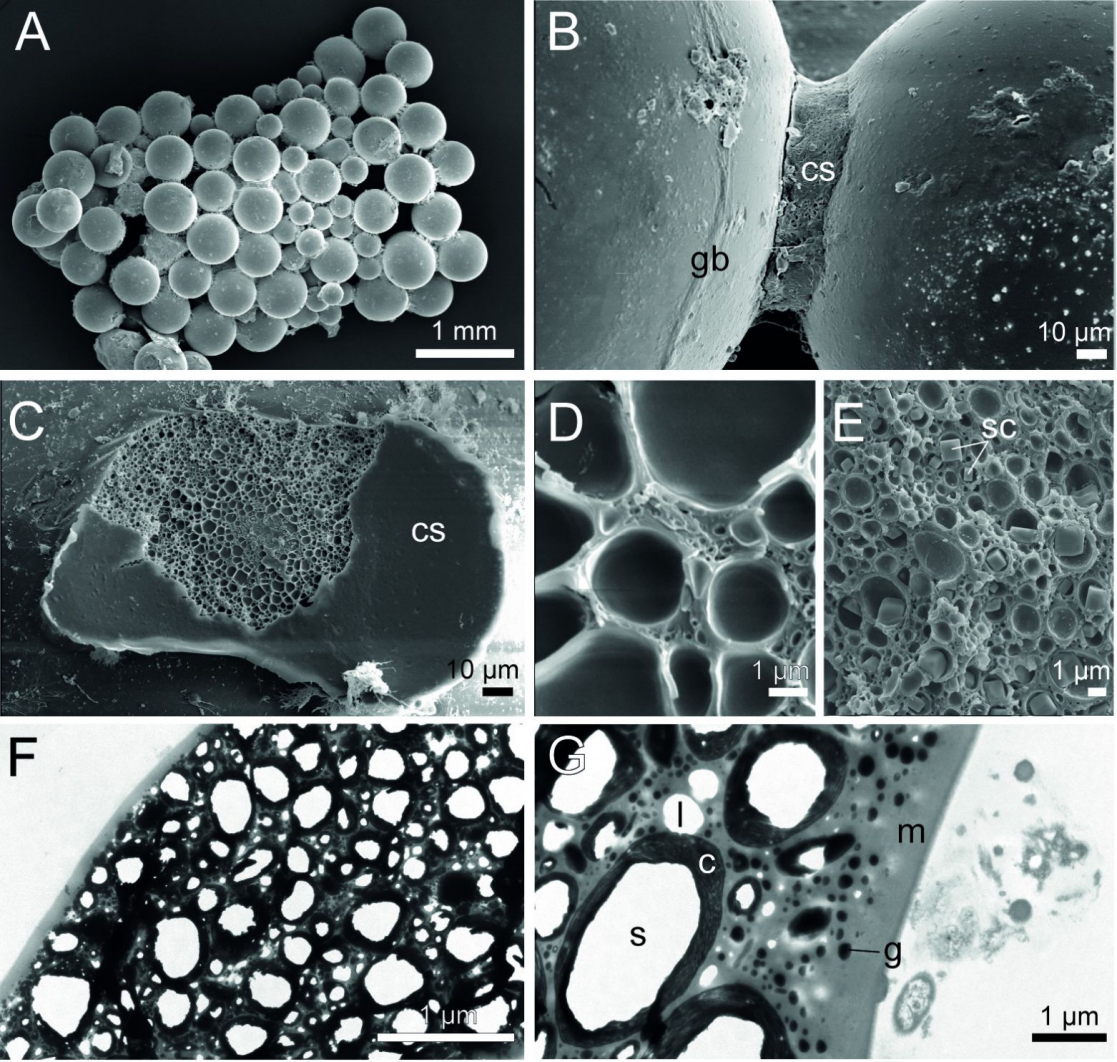
Ultrastructure of the cement in *Sabellaria alveolata*. SEM images of a glass bead tube (A), with a closer view of a spot of cement connecting two beads (B). A cohesive failure in the cement spot reveals pores of varying diameters (C–E), with some dry salt crystals found inside a pore (E). TEM images reveal the complex ultrastructure of the cement spot (F, G). Abbreviations: c – cortex; sc – salt crystal; cs – cement spot; g – electron-dense granule; gb – glass bead; l – lacunae; m – matrix; s – spheroid.

Tube reconstruction was induced by placing amputated tubes containing worms in a Petri dish filled with glass beads. Within less than 24 h, a newly formed tube made of glass beads could be observed (Figure 1E, Figure 2A).

### Ultrastructure of the cement

SEM observations of the tubes made up of glass beads show that the beads are connected one to another by four to five cement spots with diameters ranging from 100 to 160 µm (Figure 2A–C). Cement spots display a smooth outer skin both at their margin and at the interface with the glass beads, while their inner core is porous (Figure 2C–E). The pores revealed by the cohesive failure of the cement present diameters varying from approximately 0.25 to 4 µm (Figure 2C–E). Pores are larger in the center of the cement spot and decrease in size towards the edges (Figure 2C). In an unfixed, air-dried tube fragment that was subsequently broken, SEM imaging and microanalysis of a fractured cement spot showed one NaCl crystal within each of the pores (Figure 2E, Supporting Information File 1, Figure S1).

A decalcified cement spot that held two glass beads together was also observed in TEM (Figure 2F,G). The cement matrix is homogeneous and of medium electron density. It encloses hollow spheroids of various sizes, as well as small electron-dense granules and small lacunae (Figure 2F). The exception is the periphery of the cement spot, which is made up entirely of the matrix, giving it a smooth appearance. The hollow spheroids, measuring about 0.3–6.8 μm in diameter, appear empty at their centers. Their cortex is electron-dense and possesses a concentric lamellar structure. The thickness of this cortex also seems to increase with the spheroid size and can measure up to 400 nm. The sizes of the electron-dense granules and lacunae are 50–700 nm and 50–1400 nm in diameter, respectively. They are homogeneously distributed in the matrix between the hollow spheroids (Figure 2F,G).

### Morphology and ultrastructure of the adhesive glands

**Figure 3:**
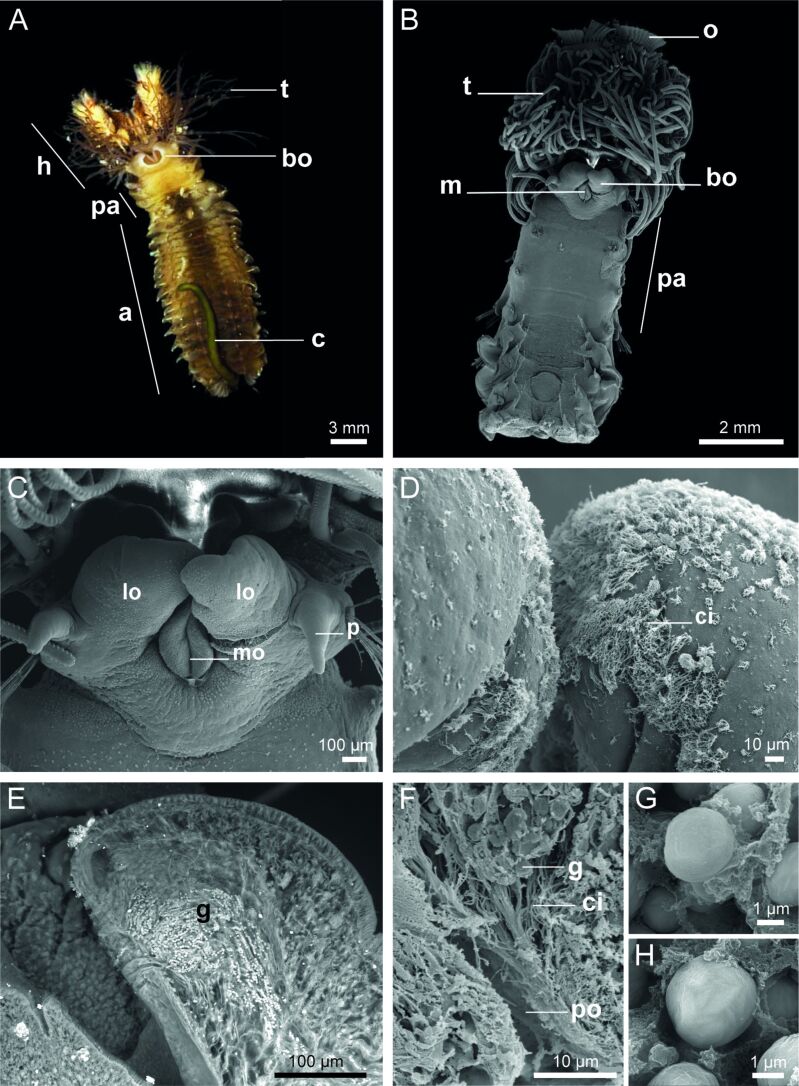
Morphology of *Sabellaria alveolata*. General ventral view of an individual (A) and SEM images showing the location and structure of the building organ (B, C). Detail of the tips of the lobes of the building organ showing cilia (D). Backscattered electron image of a longitudinal section through one building organ lobe showing secretory granules travelling towards the pit-like opening (E). Detail of the pit-like opening (F). Backscattered electron images of the two types of cement granules found in the adhesive glands: homogeneous granules (G) and heterogeneous granules (H). Abbreviations: a – abdomen; bo - building organ; c – cauda; ci – cilia; g – granule; h – head; lo - building organ lobe; mo – mouth; o – operculum; p – palp; po - pit-like opening; pa – parathorax; t – tentacle.

The body of *S. alveolata* measures approximately three to four centimeters in length and is divided into four regions, namely, the head, parathorax, abdomen, and cauda (Figure 3A). The parathoracic region comprises three segments preceding the abdomen, which forms the bulk of the body, and the cauda, an unsegmented, smooth tube terminating at the anus (Figure 3A). Anteriorly, the operculum caps approximately 250 oral tentacles involved in capturing mineral and food particles (Figure 1F, Figure 3A,B). Partially surrounding the mouth, the building organ (a horseshoe (or U)-shaped structure) is located in the thoracic area, immediately below the tentacles (Figure 1F, Figure 3B,C). Its surface is covered with cilia, which are especially numerous at the tip of the lobes (Figure 3D). A pit-like opening that allows for the release of secretory granules is visible on the inner face of each lobe, slightly below its tip (Figure 3E,F).

**Figure 4:**
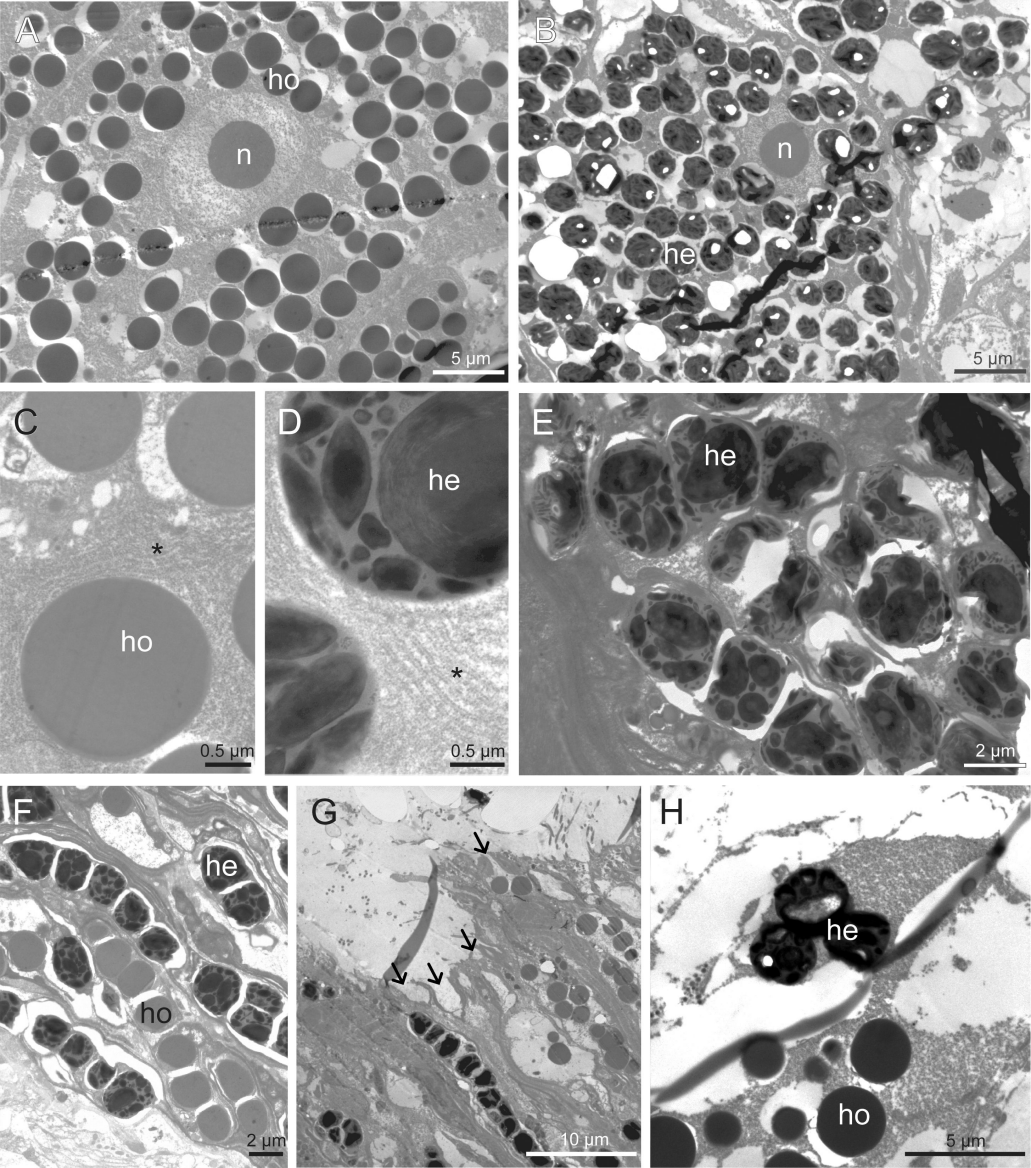
TEM images of the two types of cement cells. Cement glands with homogeneous granules (A, C) and with heterogeneous granules (B, D, E). Asterisks indicate the rough endoplasmic reticulum. The secretory granules are transported through long cell processes (F) towards the building organ where they are released through pores leading to the pit-like openings (G). Arrows indicate the pores. Granules are released intact and then gradually expand and coalesce (H). Abbreviations: he – heterogeneous granules; ho – homogeneous granules; n – nucleus.

The two lobes of the building organ form the external part of a complex secretory organ made up of clusters of cement cells located deep within the parathoracic segments of the worm [6,17]. Using transmission electron microscopy, two main types of cement cells can be distinguished based on the ultrastructure of their secretory granules, that is, cells with homogeneous granules and cells with heterogeneous granules (Figure 3G,H, Figure 4A,B). Both types of granules have a size between 2.5 and 4.0 μm in diameter. Homogeneous granules have a uniform electron density with no internal structure (Figure 4A,C). In contrast, heterogeneous granules contain conspicuous inclusions of various shapes within a matrix that is less electron-dense and resembles the contents of homogeneous granules (Figure 4B,D). These inclusions, which vary in size from 100–1500 nm, appear as spherical to elliptical and are made up of electron-dense concentric lamellae (Figure 4D). In a few samples, some inclusions, particularly the larger ones, show an apparently empty cavity in their center (Figure 4B; Supporting Information File 1, Figure S2). The granules occupy most of the cytoplasm of the cement cells, with the nucleus and the rough endoplasmic reticulum being the only visible organelles in the cell bodies. Granules also fill the cellular processes that extend up to the building organ (Figure 3E, Figure 4F). Granules are secreted through pores in the pit-like opening of the building organ, an area where epidermal cells are densely ciliated (Figure 3D,E, Figure 4G). Newly secreted granules can still be easily identified (Figure 4H). Their contents appear to gradually expand and coalesce to form a structure reminiscent of a cement spot (Figure 4H).

### Elemental composition of the secretory granules and cement

**Figure 5:**
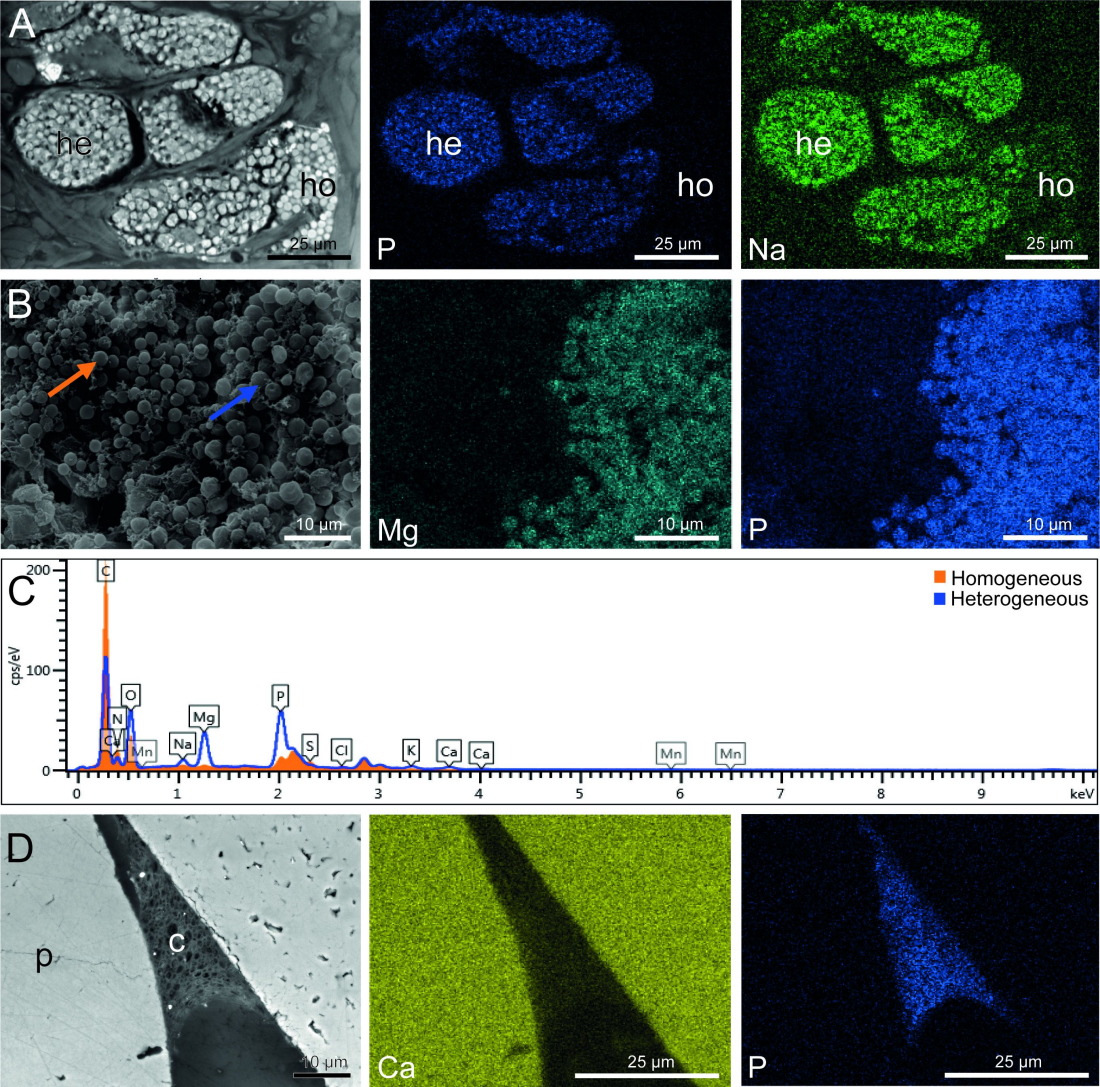
Elemental composition of the secretory granules and cement in *Sabellaria alveolata*. (A) SEM backscattered electron image with EDS spatial maps for phosphorus and sodium for the two types of cement cells. (B) SEM secondary electron image with EDS spatial maps for magnesium and phosphorus for the two types of cement cells. (C) Representative EDS spectra from the homogeneous granules (orange-filled peaks) and from the heterogeneous granules (open blue curve), as indicated in (B). (D) SEM backscattered electron image with EDS spatial maps for calcium and phosphorus for a cement spot sticking two mineral particles together in a natural tube. Abbreviations: c – cement; he – heterogeneous granules; ho – homogeneous granules; p – particles constituting the worm’s tube.

To investigate the elemental composition of cement cell granules, we used energy-dispersive X-ray spectroscopy (EDS) coupled with scanning electron microscopy (SEM). Elemental composition was measured on four secretory granules of both types of cement cells in the parathoracic part of worms embedded in Spurr resin (TEM blocks). Using the backscattered electron detector, the secretory granules could be easily distinguished. The heterogeneous granules exhibited high concentrations of phosphorus (7.3% ± 1.2%), sodium (2.6% ± 0.5%), magnesium (2.4% ± 1.0%), and calcium (0.9% ± 0.3%) (Figure 5A, Supporting Information File 1, Table S1). In contrast, the homogeneous granules (Figure 5B) presented much smaller quantities of these elements: 1.7% ± 0.4% for phosphorus, 1.1% ± 0.1% for sodium, 0.6% ± 0.2% for magnesium, and no detectable amounts of calcium (Figure 5B, Table S2). Figure 5C shows two spectra taken at the level of the heterogeneous and homogeneous granules on a transverse section of the worm’s parathorax, as indicated in Figure 5B. The P, Mg and, Na signals are mostly present in the heterogeneous granules. These observations were confirmed by the mapping of these elements over the entire samples (Figure 5A,B).

We also conducted an elemental analysis on six cement spots from natural tubes embedded in epoxy resin (Figure 5D). Our measurements revealed that, in addition to carbon and oxygen, the cement primarily consisted of calcium (15.5% ± 4.5%) and phosphorus (3.7% ± 1.1%). Additionally, we detected smaller amounts of sulfur (0.6% ± 0.1%), magnesium (0.6% ± 0.2%), and sodium (0.2% ± 0.1%) (Supporting Information File 1, Table S3). Again, the mapping of Ca and P over the entire samples confirmed the results of the spectra (Figure 5D).

### Identification of new adhesive protein and kinase candidates

It has already been suggested that *P. californica* would possess up to 26 distinct cement proteins classified into four main groups, that is, (I) GY-rich adhesive proteins, (II) H-repeat adhesive proteins, (III) SY-rich proteins, and finally (IV) a miscellaneous category of diverse proteins that do not fit into the first three groups [12,16]. The sequences from *P. californica* proteins were used as queries for BLAST searches in the transcriptome of *S. alveolata* to identify new adhesive proteins. However, as previous studies have shown, limited percentages of identity in the alignment of these proteins made it difficult to find homologues [7,31]. Despite this, one transcript encoding a protein showing similarity to Pc-5 and one transcript showing similarity to Pc-3 were identified (Table 1; Supporting Information File 1, Table S5). The adhesive protein Sa-5, encoded by the transcript comp278784_c3_seq5, has 45% identity with Pc-5 and is a H-repeat protein. This protein is polybasic, with 8.6% of its amino acid composition corresponding to histidine (Supporting Information File 1, Table S5). Its corresponding mRNA is highly expressed in the transcriptome (Table 1). Another adhesive protein was also identified, encoded by transcript comp199754_c0_seq1. This transcript was incomplete, but a full-length version (contig3247) was found in the differential transcriptome of Buffet and colleagues [18]. This protein, that we named Sa-3C, has 77% identity with Pc-3B and is unique as it starts with an SY-rich region and ends with a GY-rich region (Supporting Information File 1, Table S5). It contains a signal peptide and has a molecular weight of 38.4 kDa (Table 1). Both Sa-5 and Sa-3C are overexpressed in the worm’s parathoracic region at the mRNA level (Table 1).

**Table 1:** List of adhesive protein and kinase candidates identified in the tubeworm *Sabellaria alveolata* after transcriptomic analyses. Indicated are the name of the protein (and NCBI accession number of the transcript if available), the transcript ID from the transcriptome of the anterior part of the worm, the normalized expression level of the transcript in the transcriptome (FPKM), the differential expression of the transcript between the parathoracic part of the worm and the rest of its body (log2-FoldChange reported in Buffet et al. [18]), the amino acid length, presence of a signal peptide, the conserved domain, and the top reciprocal BLAST hit (name of the protein, species, accession number).^a^

Protein candidate	ID transcripts	FPKM	Differential expression	Length (aa)	Signal peptide	CDD	Reciprocal BLASTP hit

Sa-1 (CCD57419)	comp225468_c0_seq2	30403.9	−4.25	231	Y	NA	cement precursor protein 1 *Sabellaria alveolata* CCD57419.1
Sa-2 (CCD57440)	comp271660_c3_seq1	23866.7	−3.95	234	Y	NA	cement precursor protein 2 *Sabellaria alveolata* CCD57440.1
Sa-3A (CCD57461)	comp282003_c2_seq7	3233.6	−4.88	228	Y	NA	cement precursor protein 3A *Sabellaria alveolata* CCD57471.1
Sa-3B (CCD57482)	comp267107_c0_seq4	1867.6	−4.96	216	Y	NA	cement precursor protein 3B *Sabellaria alveolata* CCD57482.1
Sa-3C	comp199754_c0_seq1 Contig3247^b^	252	−4.07	405	Y	NA	NA
Sa-5	comp278784_c3_seq5	3880.1	−4.02	128	Y	NA	NA
SaFAM20C-1	comp253537_c0_seq2	1.3	NDE	545	Y	Fam20C-like superfamily	hypothetical protein partial mRNA *Helobdella robusta* XM_009033198.1
SaFAM20C-2	comp288995_c0_seq4	5.5	–	635	Y	Fam20C	extracellular serine/threonine protein kinase FAM20C-like *Octopus sinensis* XM_029780782.2
SaFAM20C-3	comp284991_c0_seq1	4.1	NDE	577	Y	Fam20C	extracellular serine/threonine protein kinase FAM20C-like *Octopus sinensis* XM_029780782.2
SaFAM20C-4	comp280217_c0_seq2	2.22	–	433	Y	Fam20C-like Superfamily	glycosaminoglycan xylosylkinase *Crassostrea gigas* XM_011454801.3
SaFAM20C-5	comp278295_c0_seq4	3.65	NDE	465	N	Fam20C-like Superfamily	hypothetical protein partial mRNA *Helobdella robusta* XM_009033198.1

^a^NA not applicable; NDE not differentially expressed; ^b^All the indicated parameters are for transcript Contig3247 from Buffet et al. [18], except for the FPKM value, which corresponds to the proportion of transcript comp199754_c0_seq1 within the transcriptome of the anterior part of *S. alveolata*.

In this study, we hypothesized that FAM20C kinases might be the enzymes responsible for the phosphorylation of serine residues in the adhesive proteins of the honeycomb worm. Tagliabracci et al. [21] showed that there is a high protein sequence homology in the FAM20 family across different species. A BLAST search in the transcriptome of *S. alveolata* with different FAM20C sequences from other species retrieved from the NCBI database was therefore conducted. From this analysis, five transcripts were obtained, and their translated protein sequences, named SaFAM20C-1 to -5 (Table 1), were analyzed to look for the signature amino acid features characteristic of FAM20 kinases [22]. These features include a glycine-rich loop that covers the ATP-binding pocket, a highly conserved DRHHYE motif characteristic of the enzyme active site, and another highly conserved motif (a variant motif of DFG) binding a divalent cation required for catalysis (Supporting Information File 1, Figure S3) [20,22,32]. Of the five candidates we selected, two do not meet these criteria. SaFAM20C-1 (encoded by transcript comp253537) lacks the glycine-rich loop and the DRHHYE motif (Supporting Information File 1, Figure S4). SaFAM20C-5 (encoded by transcript comp278295) does not contain a signal peptide or any features of the FAM20C enzymes (Supporting Information File 1, Figure S4). The reciprocal BLAST hits revealed similarities between these two sequences and sequences that are not described as FAM20C sequences (Table 1); they were not selected for further experiments. SaFAM20C-2 (encoded by transcript comp288995), SaFAM20C-3 (encoded by transcript comp284991), and SaFAM20C-4 (encoded by transcript comp280217) contain a signal peptide, a FAM20C domain, and the active site features of the enzyme (Supporting Information File 1, Figure S3 and Figure S4). Their reciprocal BLAST hit corresponds to sequences described as belonging to the FAM20C family, making them good candidates. However, these proteins are not differentially expressed in the parathorax of the worm (Table 1) and RT-PCR experiments showed that their corresponding mRNAs are expressed in all regions of the body (Supporting Information File 1, Figure S5).

### Localization of new adhesive protein and kinase candidates

**Figure 6:**
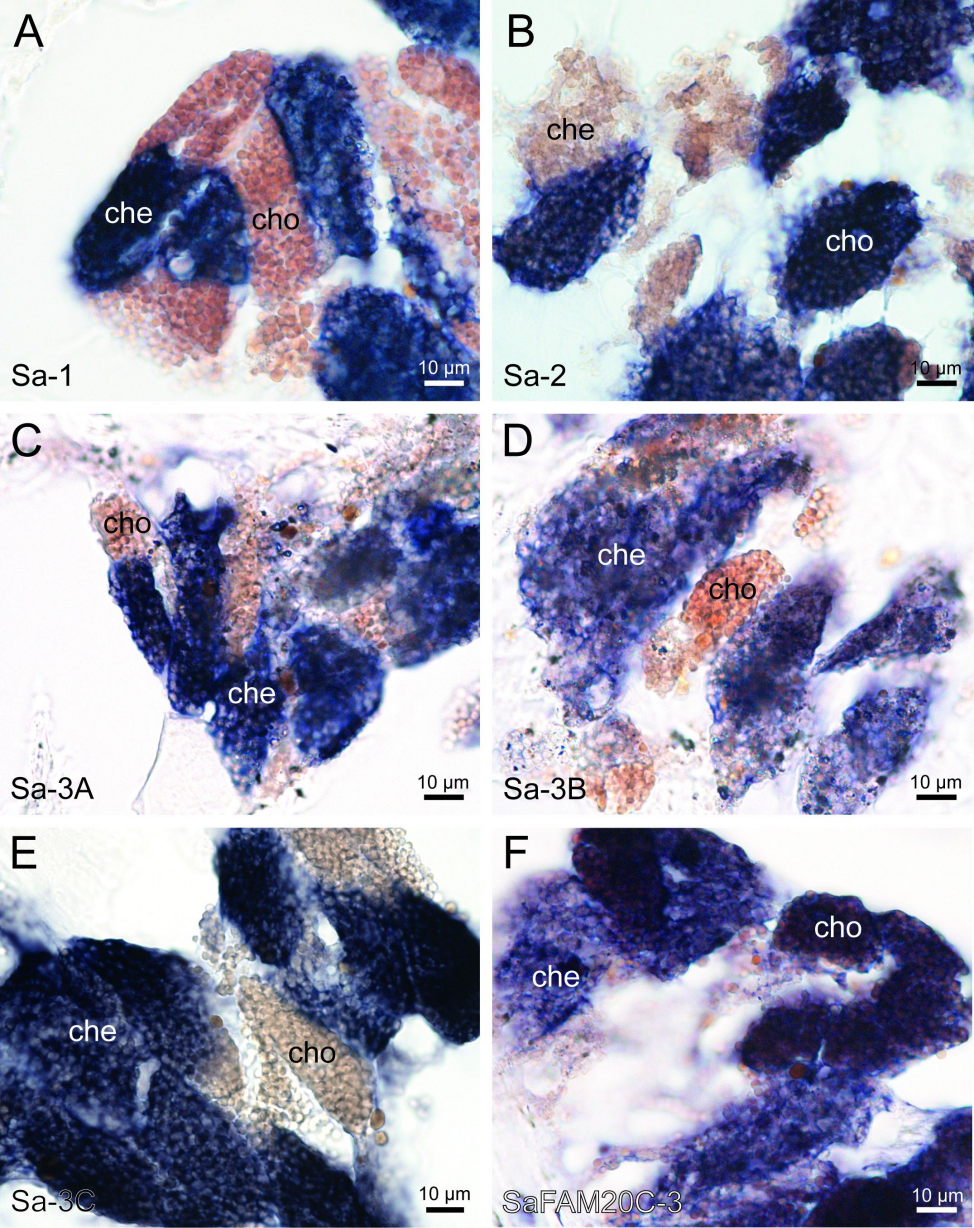
Localization of adhesive protein (A–E) and kinase (F) transcripts in *Sabellaria alveolata* using in situ hybridization. Abbreviations: che – cement cell with heterogeneous granules; cho – cement cell with homogeneous granules.

The localization of cells synthesizing the main adhesive proteins of *S. alveolata* (Sa-1, Sa-2, Sa-3A/B/C, and Sa-5) was carried out using in situ hybridization to see if this localization corresponds to that of their homologues in *P. californica*. Using DIG-labelled RNA probes, we labelled the mRNAs encoding the adhesive proteins on a section of the worm’s parathoracic part that displayed the cement cells. Control experiments were conducted with sense RNA probes, without probes, and without antibody (Supporting Information File 1, Figure S6). Sa-1 and Sa-2 proteins were expressed in cement cells with heterogeneous granules and in cells with homogeneous granules, respectively (Figure 6A,B). These expression sites are similar to those observed for Pc-1 and Pc-2. Like Sa-1, all three variants of Sa-3 were expressed in cement cells with heterogeneous granules (Figure 6C–E), consistent with the distribution pattern observed for *P. californica* [12]. Unfortunately, Sa-5 could not be localized. The localization of the three FAM20C candidates was done using the same method (Figure 6 and Supporting Information File 1, Figure S6). The results show that all the sequences are mostly expressed in both types of cement cells, but a faint signal was also detected in the epithelium of the digestive tract after a prolonged exposure time (Supporting Information File 1, Figure S6).

## Discussion

*Sabellaria alveolata* and *Phragmatopoma californica* are two tube-dwelling marine polychaetes of the family Sabellariidae. They are called honeycomb worms and sandcastle worms, respectively, because they are gregarious and the tubes of all individuals are closely imbricated to form large reef-like mounds in the intertidal zone. As they belong to sister genera [33], their adhesive systems are remarkably similar although some differences have been noted such as the absence of sulfated polysaccharides in *S. alveolata* [31].

### Production of a solid composite material forming highly resistant cement spots

**Figure 7:**
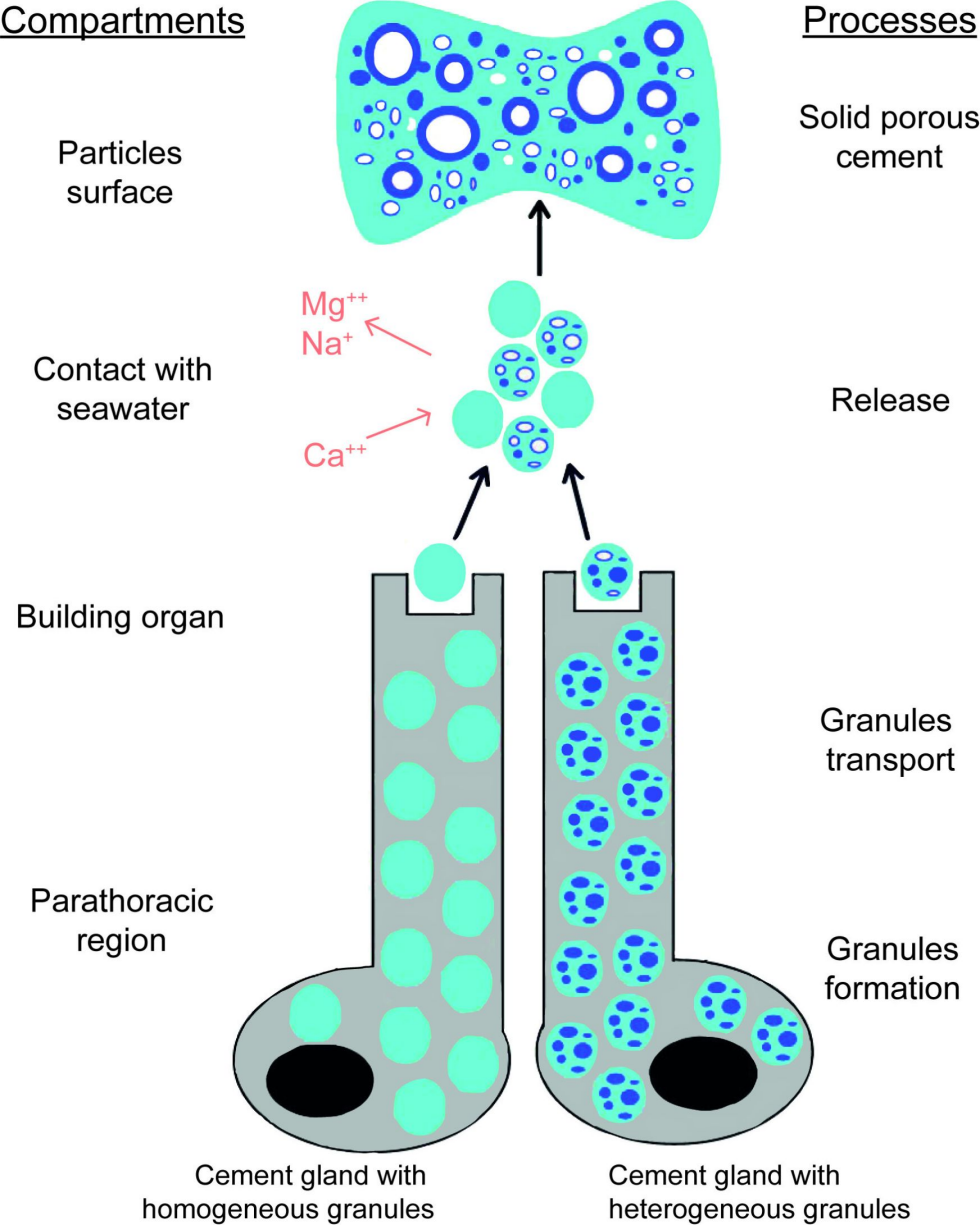
Schematic model of honeycomb worm cement formation. Granules from both types of cement cells are secreted simultaneously through individual pores, and their contents coalesce to form the matrix of the cement spot. Upon secretion, some elements might be replaced by others to facilitate complexation with the negatively charged phosphoserine residues of the adhesive proteins. Additionally, a pH shift could trigger changes in bonding, leading to spheroid hardening*.* Finally, the cement spot is cured through quinonic cross-linking.

The ultrastructural study (SEM and TEM) of the adhesive system of *S. alveolata* definitively confirms the presence of two types of cement cells in *S. alveolata*, namely, cement cells containing homogeneous granules and those containing heterogeneous granules (Figure 7). These cells are located in the three parathoracic segments, around the digestive tract and at the base of the parapodia of the honeycomb worm. Both types of granules have the same spherical shape and size, and they are very similar to those described in *P. californica* [11]. In *S. alveolata*, the use of TEM added some details. Homogeneous granules have a uniform content with no internal substructure, while heterogeneous granules contain very electron-dense inclusions formed by concentric lamellae that are embedded in a homogeneous matrix.

TEM observation of the cement spot revealed a homogeneous matrix containing three types of structures, that is, hollow spheroids of variable sizes, small dense granules, and small lacunae (Figure 7). The secretory granules from both types of cement cells are excreted simultaneously through individual pores on the epidermal surface of the building organ. There, the homogeneous material from the granules of the two types of cement cells appears to coalesce to form the matrix of the cement spot. The inclusions of the heterogeneous granules disperse in this matrix to form dense granules and hollow spheroid structures. Among these, the largest spheroids (with diameters exceeding the size of a cement gland secretory granule) appear to originate from a remarkable swelling of the inclusions of the heterogeneous granules occurring through a still unknown process. This swelling was also suggested in *P. californica* based on the similar bright appearance of heterogeneous granule inclusions and spheroid cortex under scanning electron microscopy using backscattered electrons, as well as their high phosphorus content [9]. In *S. alveolata*, TEM revealed an identical lamellar structure between inclusions and spheroids. The link between these two structures is also corroborated by simple volume calculations (i.e., the volume of the largest inclusions equals the volume of the cortex in the largest spheroids). The occurrence of expanded inclusions in some samples, although it may be an artifact of preparation, could mimic intermediate states between inclusions and hollow spheroids. Time-lapse experiments on extracted secretory granules could help decipher the mechanisms behind the expansion of heterogeneous granule inclusions [9,34].

In the cement, hollow spheroids could function as microdashpots, absorbing shocks and constraints [35], or act as stiff reinforcements in a softer matrix (see below). In both cases, the composite nature of the cement would allow *S. alveolata* to live in high-energy environments. During the secretion process, pockets of seawater could be trapped in the adhesive secretion, giving rise to the lacunae visible in TEM, as was suggested for the cement of *P. californica* [35]. Alternatively, these lacunae could be filled with a non-cross-linked adhesive protein condensate like the pores in the mussel byssal plaque [36]. This material would be dissolved during sample preparation. After secretion, intermolecular quinone bonds form between adhesive proteins, involving the oxidation of DOPA residues by catechol oxidase enzymes produced by both types of cement cells [13,15,26]. These bonds allow the adhesive to solidify within a few hours, potentially explaining the porosity gradient observed in the cement spots. Curing would prevent the spheroids from expanding further. As it likely begins at the free edge of the adhesive spot and progresses toward its center, more time is available for the formation of larger spheroids at the center of the cement spot.

### The inorganic content of the cement is modified during secretion

Both the European and Californian species have heterogeneous granules with inclusions that contain phosphorus and magnesium. In *P. californica*, the concentration of magnesium is sufficient to balance the negative charges of the phosphates [9]. This high magnesium concentration is indicative of the presence of an ATP-dependent H^+^/Mg^+^ antiporter in the granule membrane [9]. In *S. alveolata*, the heterogeneous granules also contain a significant amount of phosphorus and magnesium, but also sodium and calcium. Like in *P. californica*, the divalent ions Mg^2+^ and Ca^2+^, as well as Na^+^, can also contribute to the neutralization of negative charges in the granules. However, the composition of the granules can vary according to the fixative used, as shown by Gruet et al. [10], and this could also explain the differences reported between studies. It is worth noting that a previous study conducted on *S. alveolata* found small amounts of iron and manganese in the glands’ periphery [10]. However, these metals were not detected in this study.

The elemental analysis was also conducted on cement spots. In our samples, the phosphorus content is two-fold lower in the cement spots than in the heterogeneous granules, presumably because of the mixing of heterogeneous granules with homogeneous granules in similar quantities. We observed that the amount of calcium was 15 times higher in the cement than in the heterogeneous granules. In contrast, the magnesium and sodium content strongly decreased. Moreover, the observation of NaCl crystals in open spheroids of a fractured air-dried cement spot suggests spheroids might be filled with a solution enriched in sodium and chloride ions. All this suggests that, upon secretion, Mg^2+^ and Na^+^ might be replaced by Ca^2+^ for complexation with the negatively charged phosphoserine residues of the adhesive proteins (Figure 7). The Na^+^ ions might be released in the center of the growing spheroids. Our findings align with previous studies that have emphasized the presence of calcium and magnesium in the structure of cement spots [10,37]. Deias et al. [38] analyzed the elemental composition of the cement spots from different *S. alveolata* reef sites and found that while the concentrations of most trace elements were similar to those in seawater, those of Ca^2+^ and Mg^2+^ were significantly higher than the mean seawater composition. The elemental composition of the cement secreted by *P. californica* shows more magnesium and less calcium than what we measured in *S. alveolata* [8].

In *P. californica*, it was suggested that secretion is accompanied by a jump in pH from 5 in the secretory granule to 8.2 in seawater that could trigger a change from electrostatic to ionic bonds between divalent cations and phosphate, the effect of which would be to harden spontaneously and solidify the hollow spheroids [19]. This could explain why it is important to use an EDTA treatment for decalcification of the cement spots prior to sectioning, while the cement cells did not require decalcification despite the presence of divalent cations. SEM analyses conducted on *P. californica* revealed a distortion of the spheroids in cement spots treated with EDTA compared to untreated spots [39]. Moreover, EDTA treatment had a strong effect on the mechanical properties of the cement [39].

### Proteins involved in the adhesive system

In *S. alveolata*, the localization of the adhesive proteins Sa-1 and Sa-3 in cement cells with heterogeneous granules and of Sa-2 in cells with homogeneous granules correspond to what has been described in *P. californica* for Pc-1 to Pc-3. In the Californian species, two additional adhesive proteins (Pc-4 and Pc-5) located in the heterogeneous and homogeneous granules, respectively, have been identified, and other putative adhesive proteins (Pc-6 to Pc-26) have been reported [12,16]. By comparing all putative *P. californica* adhesive proteins with the transcriptome of the honeycomb worm, a potential Sa-5 and a new Sa-3 adhesive proteins were identified. However, no other homologues have been retrieved in the European species as there are limited percentages of identity in the alignment of their adhesive proteins with those of *P. californica*.

Sa-5 is overexpressed in the worm’s parathoracic region but it could not be localized by in situ hybridization. Its involvement in the cement therefore remains hypothetical. Sa-5 has 8.6% of its amino acid composition as histidine. It has been shown that some histidine-containing adhesive proteins may function as metal-binding proteins, as observed in the mussel byssus. Mussels actively uptake metal ions from seawater, which they then incorporate into their byssus [40]. For instance, His residues in the His-rich terminal domains of preCols, the collagenous proteins that make up over 95% of the byssal threads core, can form metal coordination cross-links with zinc ions [41–42]. In the byssal plaque, mfp-4, the protein linking the plaque to the thread, contains His-rich blocks that can form cross-links with transition metal ions, particularly copper ions [43]. These metals were not detected in this study, however.

Another candidate, Sa-3C, was also identified through the in silico analyses. This candidate has a diblock copolymer structure containing a N-terminal SY-rich region and a C-terminal GY-rich region. It was found to be expressed in cement cells containing heterogeneous granules where its block structure could help anchor the inclusions in the matrix.

### Enzymes responsible for the phosphorylation of serine residues in adhesive proteins

The occurrence of protein phosphorylation in biological adhesion has been reported in various organisms such as sandcastle worms, sea cucumbers, and mussels, and proposed to be an important component for their adhesion [19,44]. For example, mfp-5, an adhesive protein found in the mussel foot, has been shown to contain phosphoserine residues that can bind to calcareous mineral surfaces [45]. But it is in Sabellariid tubeworms that this post-translationally modified amino acid seems to be the most important. In sandcastle worms, more than 25% of the cement was found to be composed of phosphoserine [15]. In this organism, it may play a role in the condensation of the adhesive proteins in the secretory granules through complex coacervation [8]. As mentioned above, it also participates in the hardening of the spheroids through ionic bonding with calcium ions [19]. Despite the important roles of phosphorylated amino acids in adhesion, the enzymes involved in phosphorylation are not fully understood.

In a previous study, researchers attempted to identify and locate a serine kinase responsible for phosphorylating the serine residues of the Pc-3 adhesive proteins in *P. californica*, but the sequence could not be found [12]. In this study, we hypothesized that FAM20C could be the serine kinase involved in this modification. It is a secreted protein kinase that phosphorylates the polyserine motifs within secreted proteins [20]. This protein is part of the FAM20 family, which also includes FAM20A, FAM20B, and is found in both vertebrates and invertebrates with elevated protein sequence homology across different species [21–22,24]. Three FAM20C variants were retrieved from the transcriptome of *S. alveolata*, which contained the glycine-rich loop, the conserved DRHHYE motif, and the DFG motif characteristic of FAM20C kinases. To confirm their involvement in adhesive protein maturation, we localized their mRNAs using in situ hybridization on paraffin sections of the parathoracic part of the worm. All three candidates were expressed in both types of cement cells, supporting the hypothesis that these enzymes might indeed be involved in the maturation of adhesive proteins. However, these FAM20C kinases were also found to be expressed in other parts of *S. alveolata*, as shown by PCR results from other body parts of the worm (Supporting Information File 1, Figure S5). This is not unexpected as it has already been shown that FAM20C kinases are involved in a wide range of biological processes and that they generate the majority of the secreted phosphoproteome in humans, suggesting several roles for these enzymes in honeycomb worms [46]. Additional in situ hybridization experiments to localize FAM20C kinases in other parts of the worm coupled with immunohistochemical labelling using anti-pSer antibodies should allow for the identification of the secretory cells other than cement cells producing polyphosphoproteins.

The kinase candidates are expressed in both types of cement glands in *S. alveolata*, but we showed that the polyphosphoserine adhesive proteins are localized exclusively in the cells with heterogeneous granules. This raises questions about why the kinases are also present in cells with homogeneous granules of *S. alveolata*. Our elemental composition analysis of the granules of the adhesive glands revealed the presence of phosphorus in the heterogeneous granules, as expected, but a small amount of this element was also found in the homogeneous granules. Becker et al. [17] showed that anti-pSer antibodies labelled the inclusions present in the heterogeneous granules, but also the homogeneous granules, though with a lower signal. Moreover, transcriptomic analysis conducted by Buffet et al. [18] identified a large diversity of cement-related proteins, with over 68% of the overexpressed transcripts assigned to the Poly(S) category. These findings suggest that other unidentified polyphosphoproteins could be present in the homogeneous granules.

## Conclusion

The findings of this study highlight the complexity of the adhesive system in *S. alveolata* but also demonstrate the need for further research into the composition and formation of this cement. At least five different adhesive proteins are segregated between two types of cement cells, with different polyphosphoproteins and cations concentrated in the inclusions of heterogeneous granules. After secretion, these inclusions would inflate through a still unexplained process possibly involving ion exchange, to form hollow spheroids dispersed in the cement matrix. A better understanding of this complex composite material would provide valuable insights into the physical and chemical processes that underline the assembly of biological materials, which could inspire the design and fabrication of innovative hierarchical materials with diverse applications in various fields.

## Supporting Information

File 1 Detailed experimental results.

File 2 *Sabellaria alveolata* adhesive proteins and FAM20C kinases candidates identified through in silico analyses.
Complete list of *Sabellaria alveolata* adhesive proteins and FAM20C kinases candidates identified through in silico analyses. Indicated are the NCBI accession number of the transcript if available, the transcript ID from the transcriptome of the anterior part of the worm, the amino acid length, proportion of transcripts in the transcriptome, completeness of the ORF, presence of a signal peptide, molecular weight, isoelectric point, the conserved domain, the top reciprocal BLAST hit, and the amino acid composition, with color coding indicating lower or higher amino acid concentrations.

## Data Availability

All data that supports the findings of this study is available in the published article and/or the supporting information of this article.
