# Supplementary material for: The cement of the tube-dwelling polychaete Sabellaria alveolata: a complex composite adhesive material
Source: Beilstein J Nanotechnol. 2025 Nov 11;16:1998–2014. doi: 10.3762/bjnano.16.138 (PMC12621633; doi:10.3762/bjnano.16.138)
Supplement: File 1 — Detailed experimental results. [file Beilstein_J_Nanotechnol-16-1998-s001.pdf]

BEILSTEIN JOURNAL OF NANOTECHNOLOGY

Supporting Information

for

The cement of the tube-dwelling polychaete *Sabellaria alveolata*: a complex composite adhesive material

Emilie Duthoo, Aurélie Lambert, Pierre Becker, Carla Pugliese, Jean-Marc Baele, Arnaud Delfairière, Matthew J. Harrington and Patrick Flammang

Beilstein J. Nanotechnol. **2025,** *16,* 1998–2014. doi:10.3762/bjnano.16.138

Detailed experimental results

License and Terms: This is a supporting information file under the terms of the Creative Commons Attribution License (https://creativecommons.org/licenses/by/4.0). Please note that the reuse, redistribution and reproduction in particular requires that the author(s) and source are credited and that individual graphics may be subject to special legal provisions.
The license is subject to the *Beilstein Journal of Nanotechnology* terms and conditions: (https://www.beilstein-journals.org/bjnano/terms)

This PDF file includes:

Table S1, S2, S3 and S4
Figure S1, S2, S3, S4, S5 and S6

Other Supplementary Materials for this manuscript include the following:

Table S5: Complete list of *Sabellaria alveolata* adhesive proteins and FAM20C kinases candidates identified through in silico analyses. Indicated are the NCBI accession number of the transcript if available, the transcript ID from the transcriptome of the anterior part of the worm, the amino acid length, proportion of transcripts in the transcriptome, completeness of the ORF, presence of a signal peptide, molecular weight, isoelectric point, the conserved domain, the top reciprocal BLAST hit, and the amino acid composition, with color coding indicating lower or higher amino acid concentrations.

Table S1: Elemental composition (in wt %) of the heterogeneous granules present in the parathoracic part of *S. alveolata* embedded in Spurr resin**.**

**Elements**	**Hetero 1**	**Hetero 2**	**Hetero 3**	**Hetero 4**	**Mean**	**Standard deviation**
**C**	53.7	43.9	45.9	58	50.4	6.6
**O**	30.7	40.2	36.8	30.5	34.6	4.8
**P**	7.4	7.2	8.7	5.7	7.3	1.2
**Na**	2.4	3.1	2.8	1.9	2.6	0.5
**Mg**	2.6	1.1	3.4	2.3	2.4	1.0
**Ca**	0.8	1.2	1.1	0.6	0.9	0.3
**Cl**	1.1	0.7	0.7	0.4	0.7	0.3
**S**	0.8	0.6	0.6	0.4	0.6	0.2

Table S2: Elemental composition (in wt %) of the homogeneous granules present in the parathoracic part of *S. alveolata* embedded in Spurr resin.

Elements	Homo 1	Homo 2	Homo 3	Homo 4	Mean	Standard deviation
**C**	66.3	66.1	64.7	64	65.3	1.1
**O**	28.4	28.7	29.1	29.5	28.9	0.5
**P**	1.5	1.3	2	2	1.7	0.4
**S**	1.2	1.3	1.3	1.4	1.3	0.1
**Na**	1.1	1.2	1	1.2	1.1	0.1
**Cl**	0.8	0.8	1.2	1.1	1.0	0.2
**Mg**	0.5	0.4	0.7	0.7	0.6	0.2

Table S3: Elemental composition (in wt %) of six different cement spots glueing mineral particles together in a natural tube embedded in epoxy resin.

Elements	Site 1	Site 2	Site 3	Site 4	Site 5	Site 6	Mean	Standard deviation
**O**	39.6	33.0	43.2	44.2	33.1	43.6	39.4	5.2
**C**	35.2	49.4	30.2	30.3	55.2	34.5	39.1	10.6
**Ca**	17.1	13.2	20.2	19.4	8.0	15.3	15.5	4.5
**P**	5.6	2.8	3.8	3.8	2.5	3.9	3.7	1.1
**S**	0.9	0.5	0.7	0.7	0.5	0.6	0.6	0.1
**Mg**	0.7	0.4	0.6	0.6	0.3	0.8	0.6	0.2
**Na**	0.3	0.2	0.2	0.2	0.2	0	0.2	0.1
**Mn**	0.3	0.1	0.2	0.3	0	0	0.2	0.1
**Cl**	0.2	0.1	0.2	0.1	0.1	0.1	0.1	0.03
**Si**	0.1	0	0	0.1	0	0	0.1	0.1
**Al**	0.1	0.03	0.03	0	0	0	0.02	0.02

Table S4: List of primers used to generate the in situ hybridization probes.

Adhesive protein candidate	NCBI accessory number	In situ primer name	In situ FW primer sequence	In situ RV primer sequence	In situ RV primer sequence	Length in situ probe
Sa-1	HE599563	Sa-1_LPA	TCTCGCTTTGGTCTCTGCAG	Sa-1_RPA	AAGCACCACCATATCCACCC	644
Sa-2	HE599584	Sa-1_LPA	TGGTCGCATTAGCTGCAA	Sa-1_RPA	ACACCGTAA CCACCTGCAC	319
Sa-3A	HE599605	Sa-3A_LPA	TCTGCCTTGTACACTCTGAGG	Sa-3A_RPA	TGAGCTTGAACTTCTACCGC	605
Sa-3B	HE599626	Sa-3B_LPA	GAAGACTTTTGCCGTATTCG	Sa-3B_RPA	AATGATGGCTGCTAGAACTAC	647
Sa-3C	NA	Sa-3C_LPA	CCGCCTTACTATGGACGTGG	Sa-3C_RPA	CTTTGCCGTATCCGCCTTTG	775
SaFAM20C-2	NA	SaFAM20C-2_LPA	AGACCCCGCCATACAACAAG	SaFAM20C-2_RPA	CTATCCAGCGCCCTCAGATG	912
SaFAM20C-3	NA	SaFAM20C-3_LPA	AGCCAGGACGAACAGAACTG	SaFAM20C-3_RPA	GGATGTCCGAAAGCTCTTCC	805
SaFAM20C-4	NA	SaFAM20C-4_LPA	TCCGTACAATGCCTCTCGTG	SaFAM20C-4_RPA	AGTGGGGACTGGCAAAACTC	857

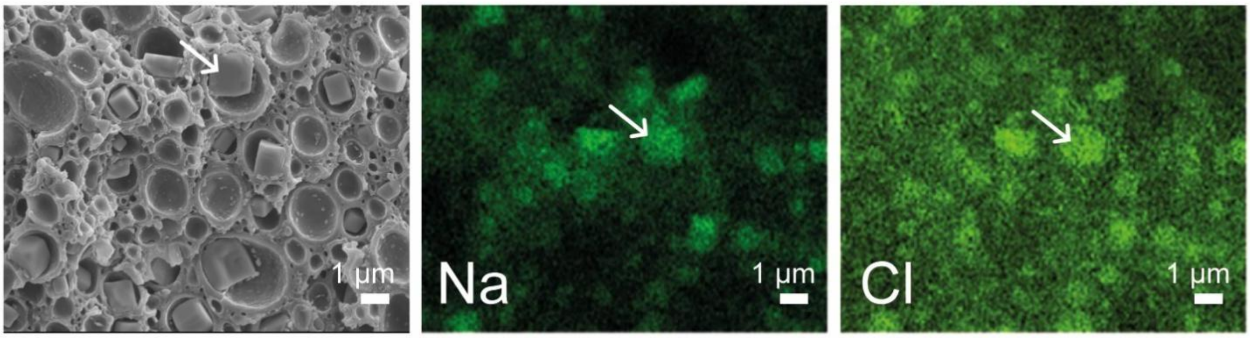

Figure S1: SEM secondary electron imaging and EDS spatial mapping of a fracture in an unfixed, air-dried cement spot from *Sabellaria alveolata* reveal NaCl crystals within the spheroids (one of the salt crystals is indicated by a white arrow).

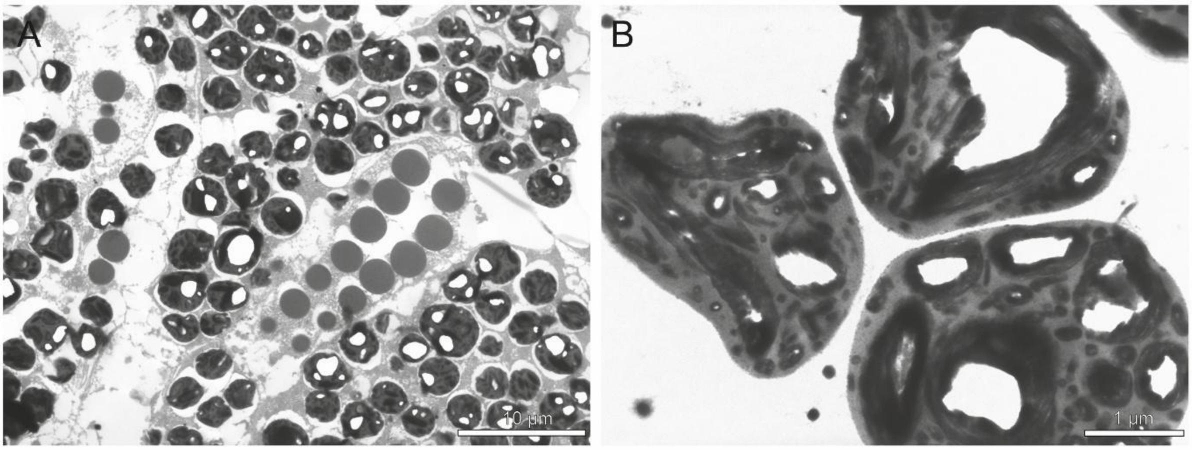

Figure S2: TEM images of cement cells with heterogeneous granules containing expanded inclusions with an apparently empty cavity at their center.

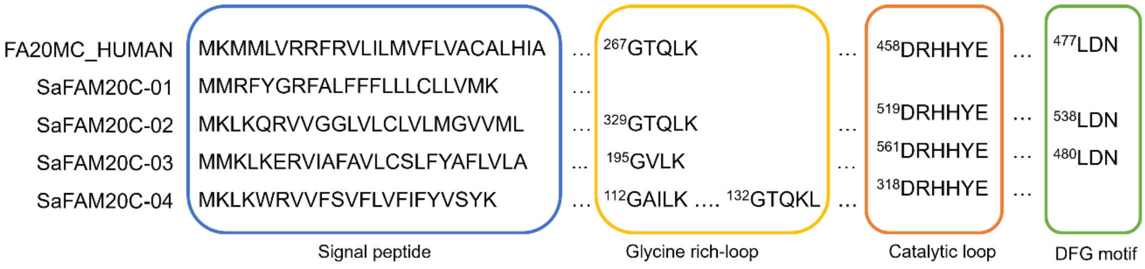

Figure S3: The four candidate SaFAM20C protein sequences aligned with the human FAM20C reference sequence (NCBI accession number Q8IXL6.2). The FAM20C signatures include the N-terminal signal peptide (blue), the glycine-rich loop (yellow), the highly conserved aspartate residue in the DRHHYE catalytic loop (orange), and the DFG-binding motif (green).

>SaFAM20C-1

MMRFYGRFALFFFLLLCLLVMKKQVITLFGTAREDTALRIVEGDTDLINKPTFIKNKENRNIESPKEGNGILQRENQV
INKFMSTEFLKPQYGSSALPDKHMEVIDELQKHIQSLNISLLEIKELLSVNSTMTASSKHRMPTRVKHINTTDNDIITR
APNDYVSSERSTVHTQATDEYFASDIVQYQKYRNIWNWANNSISHESLFPGKSPEVDTVLNALATARILSIEWFTMG
KKFYENGTSFKWIAMLEGGQKAVIKLAWEEFGGQKKGGRCNDGHELPAAEIAAFHLHRILGFYNTPYVSGRWIDLI
HEIYPVACEFVRKQITLLPNGDACVSSGILMYNDNQTLCVSGGKIKASIGYWIPRPLKLYTWYPKYAPFSMARKEWL
DIGFNNKSYCDKIPKTIKPYDQVNYYNDLFDFGLLDMLMYHFDTKHYVIDDGSSANGLTIRLDHGRAFCCYERDSM
NRVFAPIKQCCSLRKTTYERLKAFRHGNVLSLQLKSALKKDPLYPVLFEGWYSALERRLGVLFDTLEKCIQANGRD
NVLLSS

> SaFAM20C-2

MKLKQRVVGGLVLCLVLMGVVMLKGSLSFPSHLTDTDDTVLKSPHRVKPRHMDPFVRNSVRLSNDANNAGANH
MPNAYANNMPNVDAPESQMKNGLNELMGHLNVMRQELDASNYTSNDRSPEGQRVQSRQSPPSPDQQLQKLILGD
KYIMNRADDIIRLAHVIEETRAGGDVSKNDKPKEFIPNADVEFDKRSQFETGLNDDSDIPPGHEYIRIRKAKERELMA
DQVEKYEREQKQIQRMEVYILPLVMHELEDIATKMTKTRRPSHRRRGATFKIASGSIKNLTGWEKFHNHITQFDMY
DPKDPAIQQVLDDLARQPLVELYQHDGGTQLKIVYWLENDGRALFKPMRFTRDHETLPNHFYFSDYERHTAEIASF
HLDKVLDFHRVPPTAGRAVNMTQDIKMLADSKLLKTFFISPAGNVCFHGQCDYYCDSGHAICGTPDMLEGSLAAY
LPSSRYVSRKTWRHPWRRSYHKRKKAEWETDNTYCQRVRETPPYDTGRRLLDLMDMSVFDFLQGNLDRHHYETF
KDFGNYTFTMHLDNGRAFGKPRYDDLSILAPVYQCCLIRYSTFMKLVSFEQGPHLSEVIDKSMSHDMLYPILIDKHL
RALDRRVRIILKTIHRCVKKNGYQAVIQDDGF

> SaFAM20C-3

MMKLKERVIAFAVLCSLFYAFLVLAKYTNILNNTPLRSHQFSADEIFKDSKSQDAHLSMLRRELSLQKEDSGSDSLK
FDPKEALKVFILNDKVILQEADSIIQIAEILKESRNPKKQKYPEYPNNTHYHFQTYGDYHIIRKKPNFNESSNDHVAYR
KQVESEIKEVRETQWQREQRHQKRISVNLPEHVIEELEGVLKKMKENDLTVPKKTAPNLDIWHLKPGRTELRTWEV
FHNHITPFDLYKPEDPIIDKILHDLTNLPVVELYQHAGGTQIKFVYWFEHNGRALFKPMRFKRDMETLPDHYYFSDY
ERHNAEIASWHLDKVLGFHRVPPTAGRIMNITRDIQELADKDLHGTFFISPAGNLCFYGQCDYYCDSNHAVCGNPT
MIEGSMAQYLPSTNHFDRETWRHPWRRSYSRKRKAEWEVTPDYCHRVRKTPPYGSGRRLLDIMDLAVFDFLQDNL
DRHHYETFQKFGNYTALLHLDNGRAFGHPRVDDMSILAPISHCCEIRYSTLQKLIGFERGPKLSEIFDKSMSKDPLYP
LLLDKHLVAIDRRVKILLKTIYKCIAANGYKSVVKDDGF

> SaFAM20C-4

MKLKWRVVFSVFLVFIFYVSYKILEPELHQGGGRRDDGGIYDKADTGNSREFDSDIQEMPLPYNASRGVREKYLLQ
MYDLQWDRPLKEDPWKVAESWVTHRQVHPDYIPELGAILKWMSVATIQLADVGYKGTQLKMLLQLQGNIPVAFK
PKWFGRDEIIPGKAWNGADRHFGEIAGFHLNRILGLNKVPLVVGRTLDLKEEIMPVAKKELLKTFYTKGANTCFYG
KCLYCKNESTGVCGEGEFMEGAIVLWLPTKYKYQKWRHPYQRTYKEGKSARWEEDDSYCSLVVKQQELFQNGPR
LGDILDAAVYDYLILNADRHHYETMSEPWDSMLVMFDSGKSFASPHYDEESILAPLTQCCVIRKNLYERLLMLKDG
VLSKVLKDILNNDPISPVLSNLHLAAMDRRLVRILEAVADCLEKWGPEIVFINDEYR

> SaFAM20C-5

MRFRIRGIRCQVAFFVVLMGLVSVYFILFESSTLPDPSSVYFSKDELEKLILNKNIFHNVTYKSPFEAVYKNIMERQAK
NKSMWQMLHPTESPVVREEKYRKVWQWANDSISQEGLFPGDAPQADLVLKALATARIVSVGGLDMSEYESGTSR
VKWIAQLEGGQKAVIKLVWEKDEWSFSKAKNVIDGQLQSPCNAGHEMCFSEIAGWHLHRVLGFYNTPYVSGRQLS
LIEEIYPVANEAVRKQISILPGGDACITAKCYLCKYPQRLCVARGMIDASIAYWIPRPLKLYTYPPGYMPYSTPRMDR
WEHIGFNNKTYCKELRQTIEPYEKQRYYNDLFDFAVLDTLMYHYDSKHYVVDDNSKARGLTIRLDHGRAFCFFDE
DNAEIFLAPITQCCSLRKTTYSRLQELRYGDRLTSRLRSALEKDPLYPVLSEVWYPTLERRLKLIFAMLEKCIDANGL
NNVMLKVFKP

Signal peptide / Glycine-rich loop / Beta-3 / Delta-C / the catalytic loop / DFG motif

Figure S4: Amino acid sequences of the five SaFAM20C proteins retrieved from the transcriptome of *Sabellaria alveolata*. The signature motifs defining those proteins as FAM20C kinases are highlighted as follows: the signal peptide in turquoise, the glycine-rich loop in green, the Beta-3 in mallow, the catalytic loop in grey, and the DFG motif in dark teal.

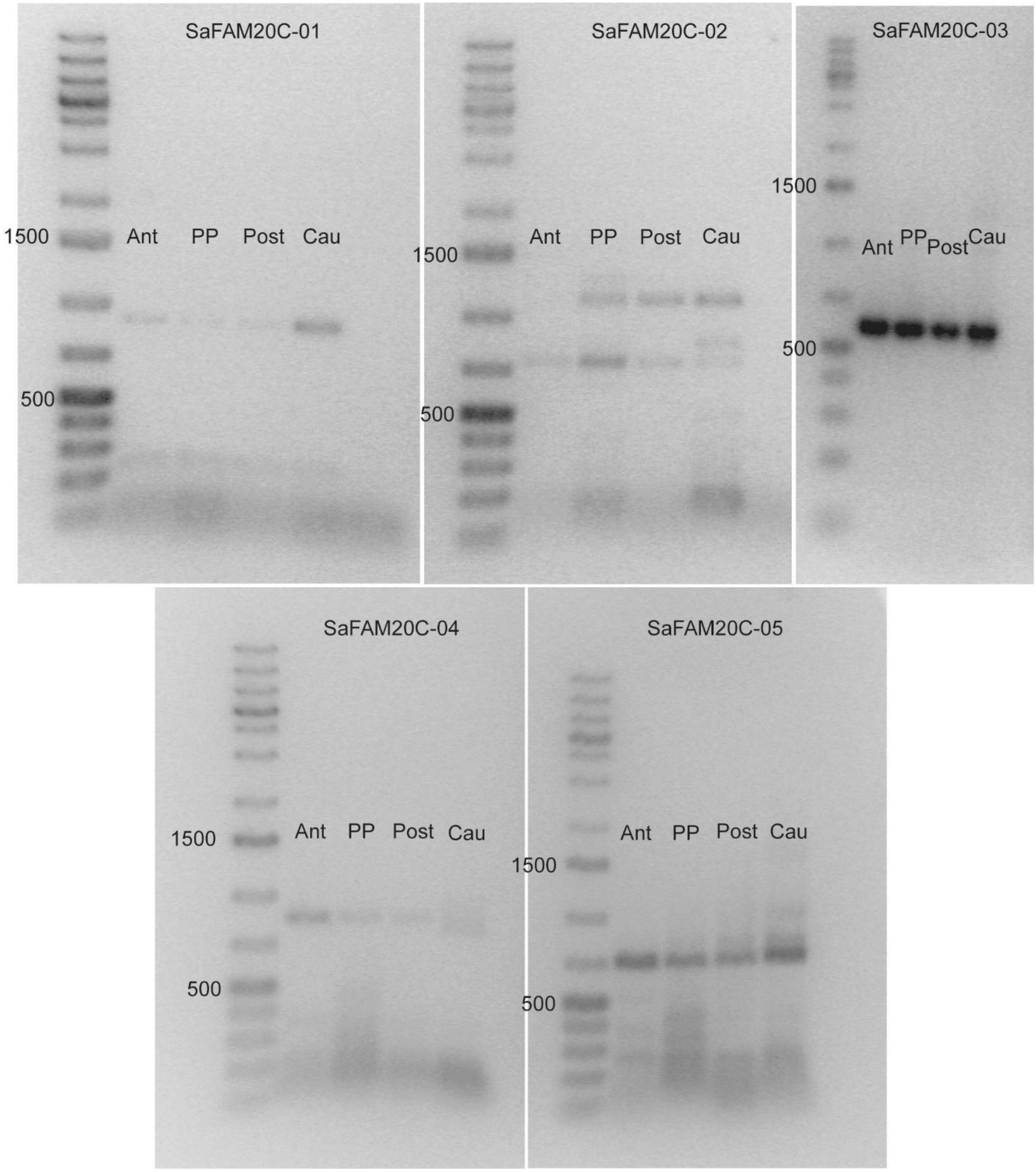

Figure S5: Body region specificity of SaFAM20C candidate genes in *Sabellaria alveolata*. RT-PCR products obtained by using transcript specific primers are separated on an agarose gel. Bands are visible in each lane, indicating the expression of SaFAM20C genes in every part of the honeycomb worm.

Abbreviations: Ant - head region; PP - parathoracic region; Post - abdominal region ; Cau - caudal region.

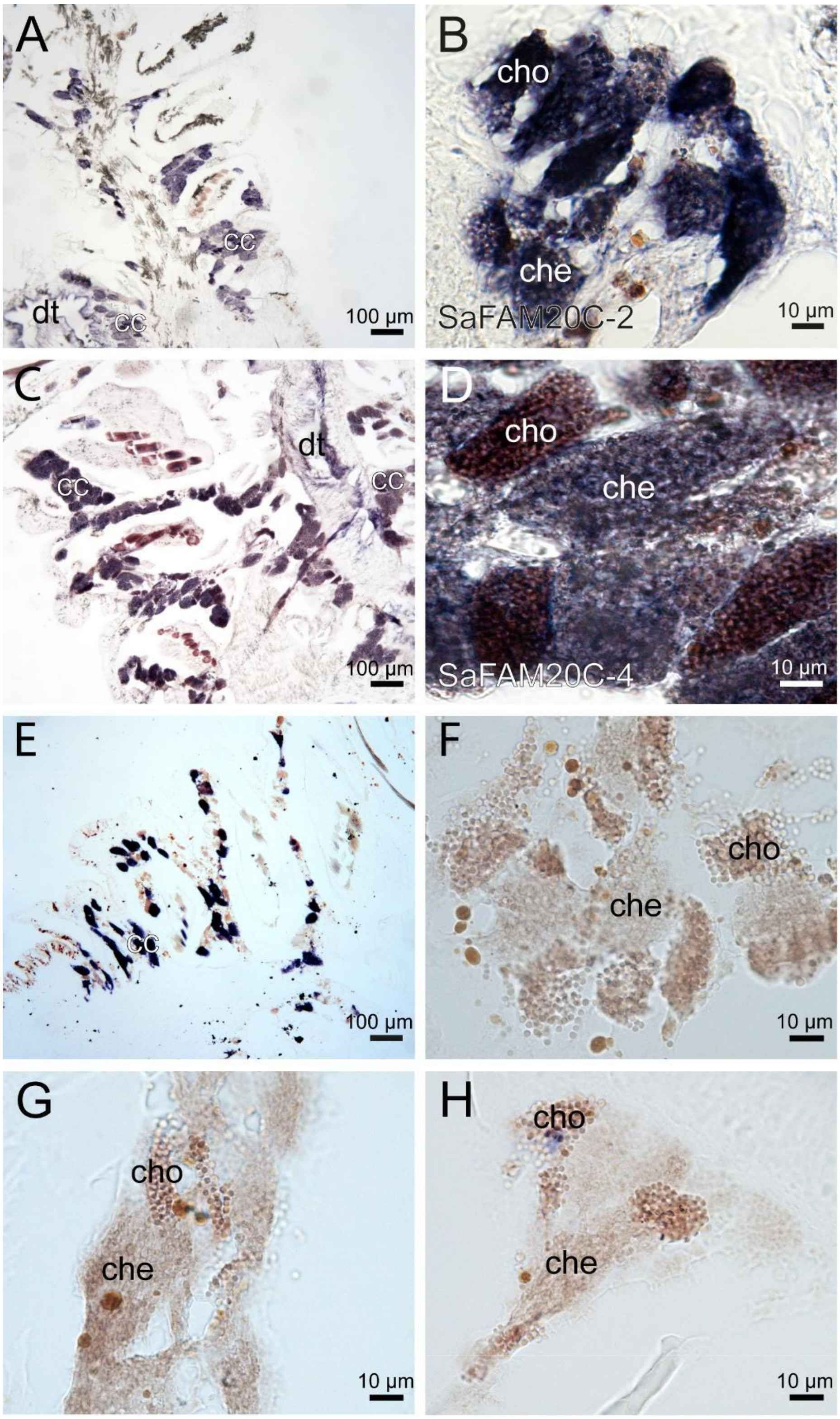

Figure S6: In situ hybridization localization of adhesive and kinase candidates in the parathorax of *Sabellaria alveolata*. Localization of the mRNAs coding for the putative kinases FAM20C-2 (A, B) and FAM20C-4 (C, D) in the cement cells. Overview image of the localization of the mRNAs coding for the adhesive protein Sa-3C (E). Negative controls without probes (F), without antibodies (G), and with sense RNA probes (H).

Abbreviations: cc – cement cells; dt – digestive tract; che – cement cell with heterogeneous granules; cho – cement cell with homogeneous granules.
